# The Effect of Antibiotic‐Cycling Strategy on Antibiotic‐Resistant Bacterial Infections or Colonization in Intensive Care Units: A Systematic Review and Meta‐Analysis

**DOI:** 10.1111/wvn.12454

**Published:** 2020-08-26

**Authors:** Xiao‐Jin Li, Yong Liu, Liang Du, Yan Kang

**Affiliations:** ^1^ Department of Critical Care Medicine West China Hospital of Sichuan University Chengdu China; ^2^ Department of Intensive Care Unit Suining Central Hospital Suining China; ^3^ Chinese Cochrane Centre West China Hospital of Sichuan University Chengdu China; ^4^ Department of Critical Care Medicine West China Hospital of Sichuan University Chengdu China

**Keywords:** antibiotic‐cycling, antibiotic‐resistant bacteria, multidrug‐resistant strains, intensive care unit, prognosis, antibiotic mixing strategy, systematic review, meta‐analysis

## Abstract

**Background:**

Antibiotic‐resistant bacteria, especially multidrug‐resistant strains, play a key role in impeding critical patients from survival and recovery. The effectiveness of the empiric use of antibiotics in the circling manner in intensive care units (ICUs) has not been analyzed in detail and remains controversial. Therefore, this systematic review and meta‐analysis were conducted to evaluate antibiotic‐cycling effect on the incidence of antibiotic‐resistant bacteria.

**Methods:**

We searched PubMed, Embase, the Cochrane Central Register of Controlled Trials, and Web of Science for studies focusing on whether a cycling strategy of empiric use of antibiotics could curb the prevalence of antibiotic‐resistant bacteria in ICUs. The major outcomes were risk ratios (RRs) of antibiotic‐resistant infections or colonization per 1,000 patient days before and after the implementation of antibiotic cycling. A random‐effects model was adopted to estimate results in consideration of clinical heterogeneity among studies. The registration number of the meta‐analysis is CRD42018094464.

**Results:**

Twelve studies, involving 2,261 episodes of resistant infections or colonization and 160,129 patient days, were included in the final analysis. Based on the available evidence, the antibiotic‐cycling strategy did not reduce the overall incidence of infections or colonization with resistant bacteria (RR = 0.823, 95% CI 0.655–1.035, *p* = .095). In subgroup analyses, the cycling strategy cut down the incidence of resistant bacteria more significantly than baseline period (*p* = .028) but showed no difference in comparison with mixing strategy (*p* = .758).

**Linking Evidence to Action:**

Although the cycling strategy performed better than relatively free usage of antibiotics in the baseline period on reducing resistant bacteria, the cycling strategy did not show advantage when compared with the mixing strategy in subgroup analyses. In addition, these viewpoints still need more evidence to confirm.

## Introduction

The emerging of resistance is a natural evolutionary process for bacteria, but it is accelerated by the selective pressure imposed by widespread usage of antibiotics. In 2014, the World Health Organization (WHO) claimed the primary species of bacteria such as *Escherichia coli*, *Klebsiella pneumonia*, and *Staphylococcus aureus* presented significant resistant to antibiotics in at least 50% of isolates (WHO, 2014). The WHO also warned if no appropriate measures were taken, more patients would face life‐threatening resistant bacterial infection. There are many factors that can lead to severely ill patients more likely to be infected with drug‐resistant bacteria, such as long‐term hospital stay, invasive operation, compromised immunity, and enrichment of antibiotic‐resistant bacteria in the environment. The prevalence of antibiotic‐resistant bacteria in ICU not only increases the mortality and morbidity of patients, but also increases the length and cost of hospitalization. Furthermore, additional isolation wards, medical materials, and medical staff are needed to limit the spread of antibiotic‐resistant bacteria (Vallés et al., 2014; Vincent et al., 2009). There have been no new classes of antibiotics discovered since 1987 (Graham, 2017). The lack of new drugs and overuse of antibiotics make the threat of antibiotic‐resistant bacteria become increasingly serious, which also underlines the importance of rational use of antibiotics for reducing the selection pressure on microbes (Xiao et al., 2013).

**Figure 1:**
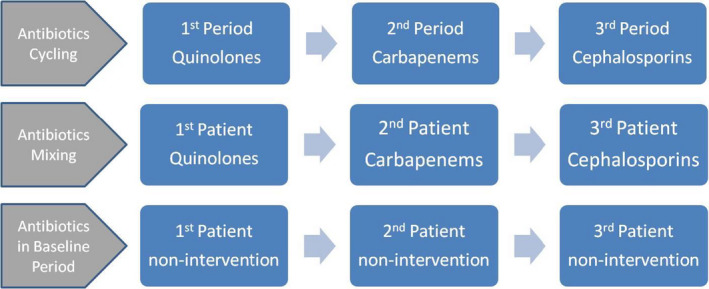
The different approaches of antibiotic using.

Many strategies have emerged to control the emergence or dissemination of antibiotic‐resistant bacteria, such as hand hygiene, chlorhexidine bath, restriction of ineffective antibiotics, reducing treatment duration, antibiotic‐cycling strategy, and antibiotic mixing strategy (Baur et al., 2017). In the cycling strategy (also called rotation strategy), a specified antibiotic is empirically used as a preferred option during a scheduled period in patients whose pathogenic microorganisms are unidentified. After that period, another antibiotic, usually from a different kind of antibiotics, becomes the first choice for all patients needing anti‐bacterial treatment. In the mixing strategy, the first‐line antibiotic will alternate in consecutive patients according to a pre‐established protocol. To help make sense of this, a schematic of approaches of antibiotic using is shown in Figure 1 with quinolones, carbapenems, and cephalosporins as examples. The two measures are believed to increase the diversity of antibiotics compared with non‐interventional (or in baseline period) antibiotic prescriptions, where antibiotic for empirical use is decided by the attending physician. It is generally assumed that different classes of unrelated antibiotics were used in turns to remove the resistance of bacteria by random loss of resistance genes from bacterial flora (also called drift). The study by Christiane and colleagues showed that alternating structurally similar antibiotics could restore the susceptibility of resistant bacteria to antibiotics (Goulart et al., 2013).

Although most of the published studies suggested that the cycling strategy was an effective approach to curb the emergence of resistant strains (Abel zur Wiesch, Kouyos, Abel, Viechtbauer, & Bonhoeffer, 2014), the opinion was challenged as some new evidence has come to light. And the new emerging studies showed that the cycling strategy about using antibiotics did not present more advantages than the mixing strategy (Cobos‐Trigueros et al., 2016; Van Duijn et al., 2018). Therefore, we systematically reviewed relevant literature and analyzed data to clarify the effect of cycling regimens on suppressing the incidence of resistant bacteria, as well as the mortality, nosocomial infection, and ventilator‐associated pneumonia (VAP) related to ICU patients.

## Methods

### Search Strategy

We searched PubMed, Embase, the Cochrane Central Register of Controlled Trials, and Web of Science from the establishment of databases to April 20, 2018, to identify potentially eligible studies evaluating the effect of an antibiotic‐cycling strategy on the acquisition of resistant bacteria. The study type, language, and publication status of targeted articles were not limited in our retrieval scheme. Search terms mainly included “antimicrobial*,” “antibiotic*,” “resistan*,” cycling, rotation, and scheduled (Table S1). To thoroughly identify the literature associated with cycling strategies, we did not add search terms such as “intensive care unit” and “ICU” in our retrieval queries. We continued to receive updated information from the databases until the review of this publication. All reference lists of articles related to antibiotic‐cycling strategy were reviewed to explore the possible eligible literature, and the studies’ authors were contacted to supplement missing information if needed. The meta‐analysis was registered on www.crd.york.ac.uk/prospero with the registration number CRD42018094464 without a published protocol.

### Inclusion Criteria and Data Extraction

The potential qualified studies were included with relevant criteria as follows: (1) critically ill patients as research subjects; (2) receiving antibiotic‐cycling strategy; (3) having control period or control group; (4) providing data of resistant bacteria infections or colonization; and (5) with before–after or cluster‐randomized study designs.

Studies were excluded for the following reasons: (1) did not provide enough data; (2) was not conducted in an intensive care unit; (3) used an unrepresentative cycling strategy; (4) included patients with hematological diseases as study subjects; (5) included patients less than 18 years old; and (6) did not include a control period or control group. Two authors independently screened the literature and extracted data from eligible studies according to a predesigned Excel form.

The data relevant to results and characteristics of the studies were extracted from eligible articles, such as publication year, country, study design, medical setting, mixing or baseline as control period, infection or colonization, total cycling duration, each cycling period length, research quality, types of antibiotics, and incidence of antibiotic‐resistant bacteria.

Disagreements between the authors were resolved by consulting a third author. Some studies provided both resistant‐infection and resistant‐colonization data, and we merged risk ratios (RRs) using the incidence of antibiotic‐resistant bacteria colonization per 1,000 patient days, considering the larger sample size for colonization than infection. If the total patient days for an ICU stay were not available, total patient days were calculated by the number of patients multiplied by the average duration of ICU stay. We chose the mixing period as the control period if a study contained both a baseline period and mixing period concerning the empirical use of antibiotics. To explore heterogeneity sources, the RRs of acquisition of gram‐positive resistant bacteria (RG+) and gram‐negative resistant bacteria (RG−) were treated as independent data in the meta‐regression analysis.

### Major and Secondary Outcomes

The main purpose of the study is to evaluate whether the cycling strategy is a more valid measure to curb the incidence of resistant bacteria than control period, the latter includes baseline period (using antibiotic without a schedule) and the mixing strategy period. Considering before–after design with a washout period being adopted relevant to antibiotic stewardship researches, we think it is reasonable to use RRs to compare data between groups. The major outcomes were RRs of incidences of antibiotic‐resistant infections or colonization per 1,000 patient days before and after implementation of antibiotic cycling, including infections caused by RG‐ and RG + resistant bacteria. Secondary outcomes included RRs of ICU mortality, hospital mortality, ventilator‐associated pneumonia, and nosocomial infection.

### Quality Evaluation

The quality of research was assessed independently by two authors (YL and XJL) using the National Institutes of Health’s Quality Assessment Tool for Before‐After (Pre–Post) Studies With No Control Group (Baur et al., 2017; National Institutes of Health, 2016). Twelve items were used to evaluate the included studies (Table S2). Studies were classified as high quality (more than eight points), moderate quality (seven to eight points), or low quality (fewer than seven points) in accordance with the results of a discussion among all authors.

A funnel plot was conducted to evaluate publication bias. In a funnel plot, the ordinate values represent the effect size of studies, and the abscissa values represent the sample size (or precision) of studies. Standard errors of the effect are smaller along with increasing sample size, and the points will locate the upper area of the funnel plot. Thus, data from included studies are expected to be symmetrically distributed in a funnel shape if there is no significant publication bias (Egger, Davey Smith, Schneider, & Minder, 1997).

The meta‐analysis was conducted following the Preferred Reporting Items for Systematic Reviews and Meta‐Analyses (PRISMA) guidelines (Moher, Liberati, Tetzlaff, Altman, & PRISMA Group, 2009; Table S3).

### Statistical Analyses

The inverse variance method was used to estimate the overall relative risk of pooled data through the random‐effects model owing to the significant clinical heterogeneity among studies. Also, the *I* ^2^ statistic was used to quantitatively describe statistical heterogeneity, with the values of 0%, 25%, 50%, and 75% were described as no, low, moderate, and high observed heterogeneity, respectively (Higgins, Thompson, Deeks, & Altman, 2003). Subgroup analyses were performed to explore the sources of heterogeneity, as well as important factors that might affect the outcome. After detailed discussion among authors, eight factors were chosen as variables to classify studies, including publication year, cycling duration, cycling length, quality, location, prospective/retrospective design, type of ICU, and control period. Also, these variables relevant to heterogeneity were further explored through meta‐regression (Higgins & Thompson, 2004). All statistical analyses were carried out using Stata 13.0.

## Results

### Study Selection

**Figure 2:**
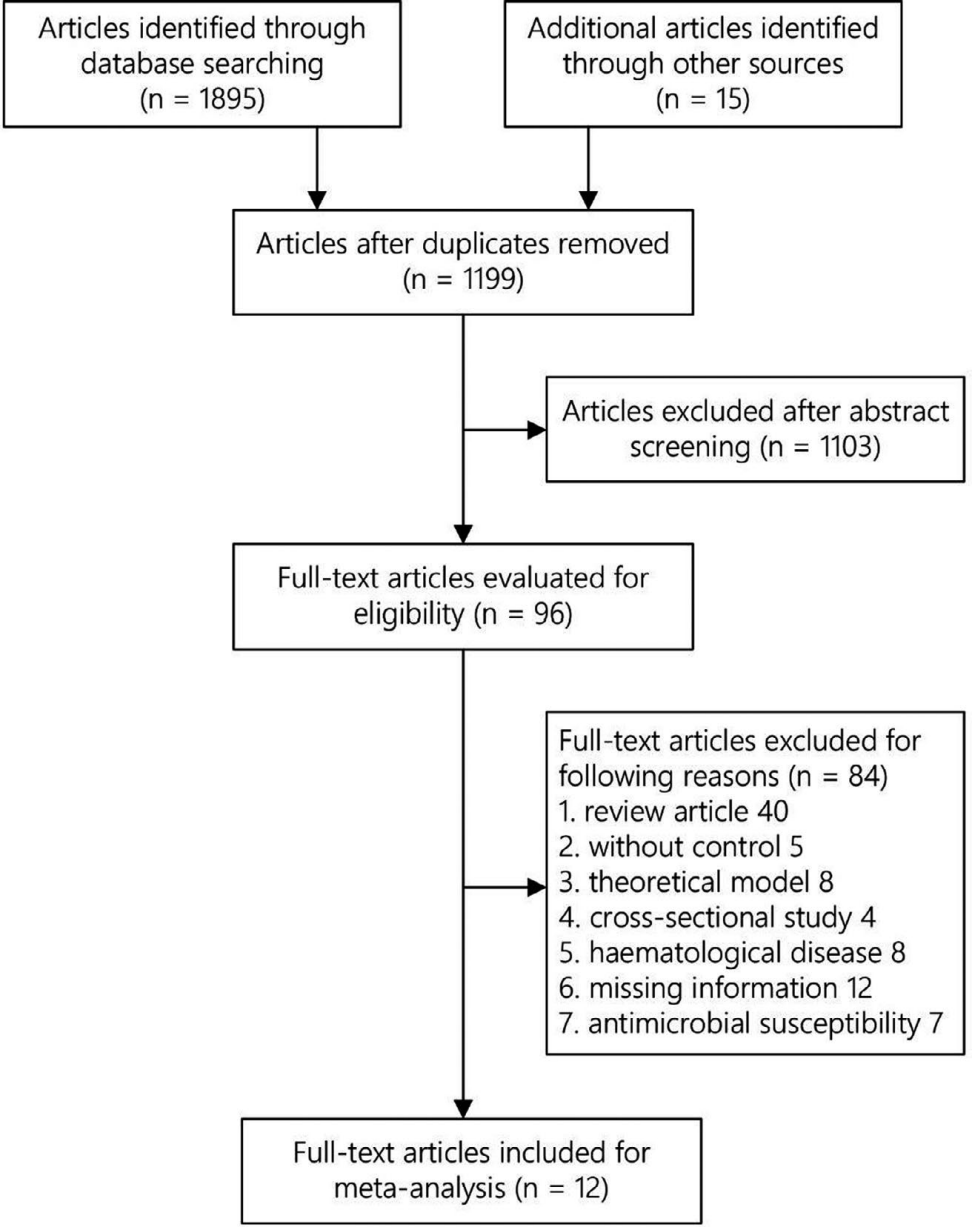
Flow diagram of study selection.

Our search scheme yielded 1895 citations, and an additional 15 citations were identified by examining reference lists of the relevant literature. Ninety‐six articles with full text were obtained after removing duplicates and reviewing abstracts, and 12 of them were included in the final analyses (Chong et al., 2013; Cobos‐Trigueros et al., 2016; Cumpston et al., 2013; Dominguez, Smith, Reed, Sanders, & Sanders Jr, 2000; van Duijn et al., 2018; Evans et al., 2005; Gruson et al., 2000, 2003; Hedrick et al., 2008; Kontopidou et al., 2013; Martínez et al., 2006; Nijssen et al., 2010; Raineri et al., 2010; Raymond et al., 2001; Sandiumenge et al., 2006; Smith et al., 2008; Teranishi et al., 2017; Toltzis et al., 2002; Warren et al., 2004; Figure 2).

### Study Characteristics

The twelve studies were collected with the publication time being from 2,000 to 2018, comprising 2,261 isolates of resistant bacteria and 160,129 patient days (Table S4). Five studies were conducted in the United States, and seven studies were conducted in European countries including France, Italy, the Netherlands, and Spain. Ten of the 12 included studies used a before–after design, and the other two used a crossover design; eight of the studies were prospective, with the study by Van Duijn et al. using a cluster‐randomized, crossover design (Van Duijn et al., 2018). The use of a mixing strategy, which means a different class of antibiotics was prescribed to each consecutive patient, served as the control period in four studies. The cycle for each antibiotic involved in a cycling strategy ranged in duration from 0.25 to 4 months. Four studies were of low quality, four were of moderate quality, and four were of high quality, according to the quality assessment tool for before–after studies described in the methods section.

### Synthesis of the Results

**Figure 3:**
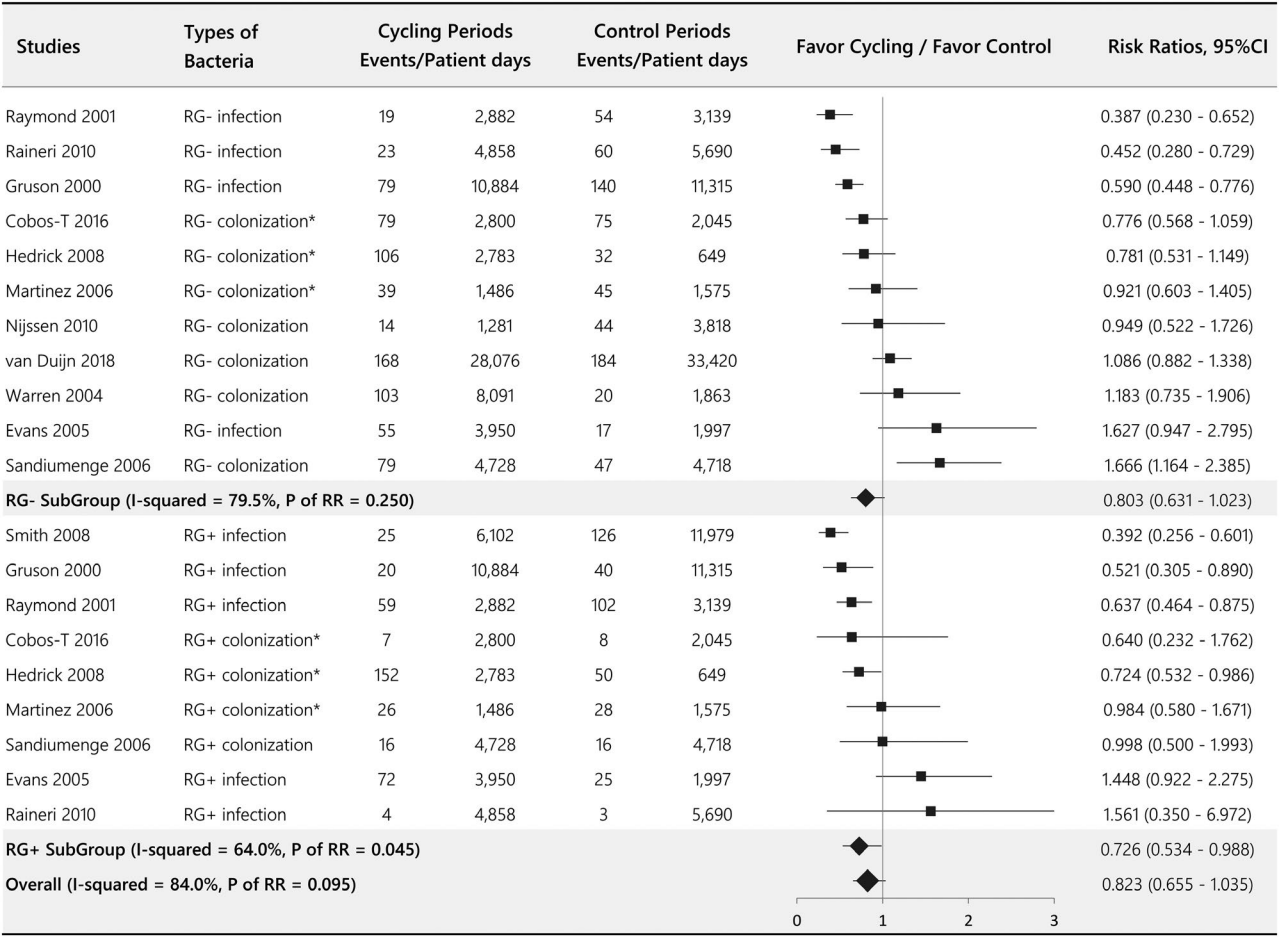
Forest plot evaluating the effect of antibiotic‐cycling on the incidence of RG+ and RG−. *The colonization, rather than infection, data were chosen for analysis. RG+ = gram‐positive resistant bacteria; RG‐ = gram‐negative resistant bacteria; RR = risk ratio.

**Figure 4:**
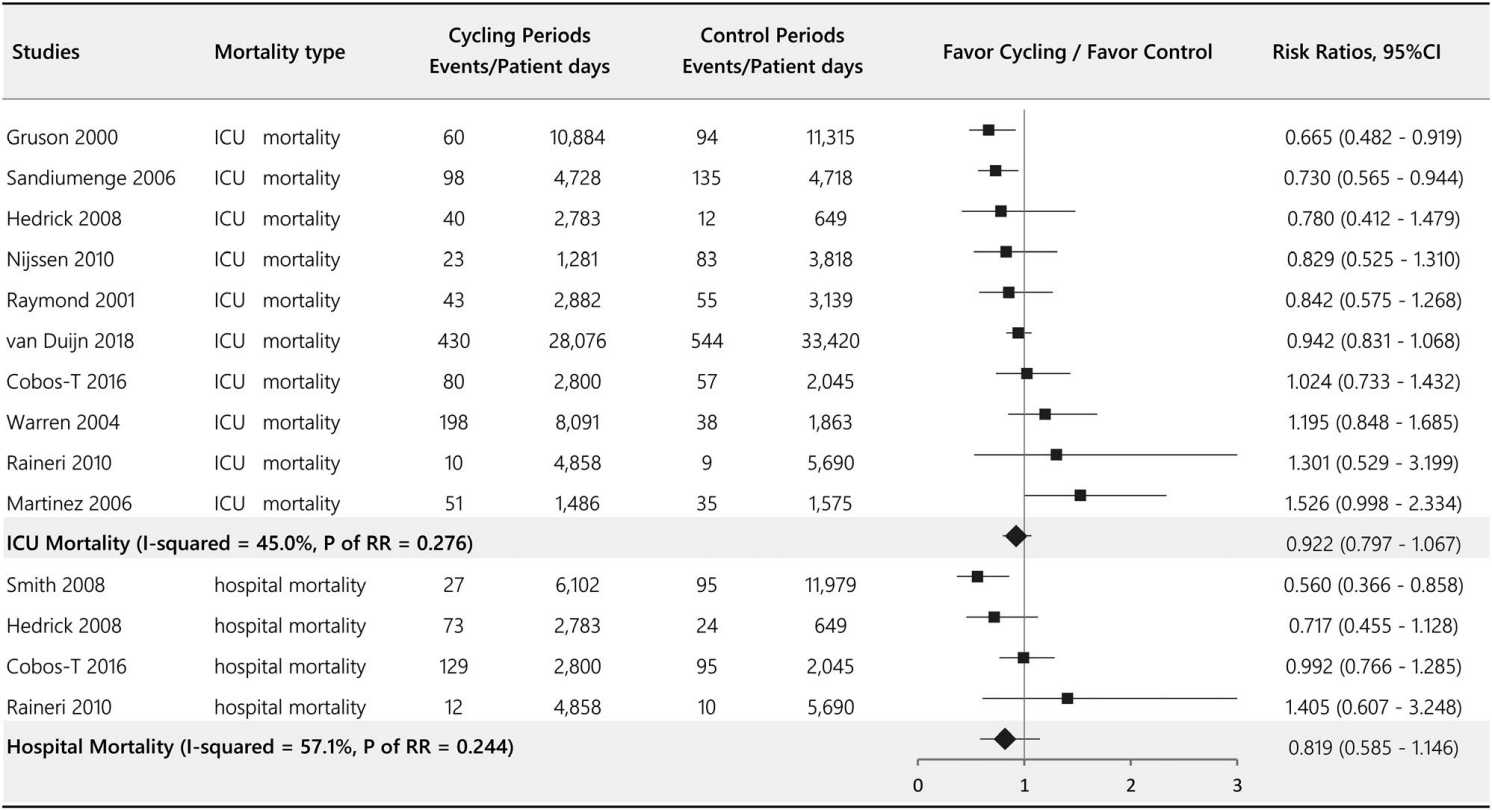
Forest plot evaluating the effect of antibiotic cycling on ICU mortality and hospital mortality.

The effects of an antibiotic‐cycling strategy on reducing gram‐negative resistant isolates were evaluated in 11 studies, with pooled estimates showing no significant difference (RG‐ = RR = 0.803, 95% CI 0.631–1.023, *p* = .250, *I* ^2^ = 79.5%; Figure 3). Nine studies assessed the effect of cycling strategy on gram‐positive resistant bacteria, and the result of the pooled analysis was favorable for the cycling strategy (RG+ = RR = 0.726, 95% CI 0.534–0.988, *p* = .045, *I* ^2^ = 64.0%). The overall pooled result after merging the RG‐ and RG + data did not present a statistically significant difference (RR = 0.823, 95% CI 0.655–1.035, *p* = .095, *I* ^2^ = 84.0%). We also collected data on the changes in common resistant bacteria affected by a cycling strategy. *Pseudomonas aeruginosa* and MRSA showed significant downward trends, but there were no differences among the resistant bacteria examined, including *Acinetobacter baumannii*, *Clostridium difficile*, *Escherichia coli*, ESBL, *Klebsiella pneumoniae*, *Stenotrophomonas maltophilia,* and VRE (Figure S1). The ICU mortality examined in 10 studies showed no significant change after implementation of antibiotic cycling (RR = 0.922, 95% CI 0.797–1.067, *p* = .276, *I* ^2^ = 45.0%), and we also did not find a statistically significant difference in hospital mortality (RR = 0.819, 95% CI 0.585–1.146, *p* = .244, *I* ^2^ = 57.1%; Figure 4). The pooled RR of nosocomial infections from 8 studies showed a downward trend during the cycling period (RR = 0.845, 95% CI 0·761–0.938, *p* = .002, *I* ^2^ = 28.2%), as did ventilator‐associated infections (VAP) assessed in 6 studies (RR = 0.757, 95% CI 0.637–0.901, *p* = .002, *I* ^2^ = 19.9%; Figure S2).

### Subgroup Analyses

Subgroup analyses were conducted based on the different characteristics of the included studies (Table 1). When grouped according to publication year, we found that the acquisition of resistant bacteria was reduced more clearly in the 2000–2004 subgroup than in the 2005–2008 and 2010–2018 subgroups (RR = 0.683, *p* = .040. vs. RR = 0.923, *p* = .710; RR = 0.810, *p* = .215, respectively). The acquisition of resistant bacteria showed a noteworthy decrease in the medical ICU subgroup (RR = 0.784, *p* = .025) but not in the mixed and surgical ICU subgroups (RR = 0.852, *p* = .447; RR = 0.775, *p* = .706, respectively). In contrast to the studies using the baseline period as a control, the findings from studies using antibiotic mixing (a different class of antibiotics was prescribed to each consecutive patient) as a control did not show the effectiveness of the cycling strategy (baseline = RR = 0.723, *p* = .028 vs. mixing = RR = 1.041, *p* = .758).

**Table 1:** The Effect of Antibiotic‐Cycling on the Incidence of Resistant Bacteria in Accordance With Subgroup Analyses

Subgroups	Number of studies	I‐squared%	*p*‐Value of RR	Weight %	Risk ratios, 95% CI
Publication year					
2000–2004	3	75.2	.040	25.5	0.683 (0.476–0.982)
2005–2008	5	88.5	.710	42.6	0.923 (0.604–1.409)
2010–2018	4	72.0	.215	31.9	0.810 (0.581–1.130)
Cycling duration					
3–8 months	3	0.0	.132	23.5	0.854 (0.695–1.049)
12 months	4	92.0	.721	33.6	0.898 (0.497–1.622)
18–24 months	5	86.0	.089	43.0	0.741 (0.525–1.047)
Cycling length					
0.25–1.5 months	5	75.2	.190	42.3	0.835 (0.637–1.094)
3–4 months	7	88.6	.277	57.7	0.811 (0.555–1.184)
Quality					
5–6 points	4	64.3	.097	31.9	0.782 (0.586–1.046)
7–8 points	4	89.6	.318	32.0	0.739 (0.409–1.337)
9–10 points	4	88.7	.641	36.1	0.910 (0.611–1.355)
Location					
USA	5	87.9	.252	41.6	0.780 (0.511–1.192)
Europe	7	82.0	.280	58.4	0.856 (0.647–1.134)
Prospective or not					
Not prospective	4	78.9	.143	28.3	0.675 (0.399–1.142)
Prospective	8	85.9	.380	71.7	0.892 (0.690–1.152)
Type of ICU					
Medical	5	60.1	.025	43.2	0.784 (0.634–0.970)
Mixed	5	87.4	.447	40.8	0.852 (0.563–1.228)
Surgical	2	95.7	.706	16.0	0.775 (0.205–2.294)
Control period					
Baseline as control	8	82.4	.028	64.4	0.723 (0.541–0.966)
Mixing as control	4	69.1	.758	35.6	1.041 (0.807–1.342)

### Meta‐Regression and Publication Bias

A meta‐regression based on the data from RG + and RG‐ infection or colonization was performed to explore sources of heterogeneity, and we found heterogeneity among studies possibly caused by methodological quality (*p* = .008), type of ICU (*p* = .019) and the mixing strategy as a control (*p* = .030). Publication bias, including an evaluation of the effect from studies with small sample sizes, was not identified by Egger’s test (*p* = .952) or funnel plot (Figure S1).

## Discussion

### Literature Review

This review systematically assessed the impact of an antibiotic‐cycling strategy on the acquisition of resistant bacteria. The results suggested that the overall acquisition of resistant bacteria and ICU mortality did not reach statistical significance after the cycling strategy being introduced. A previous meta‐analysis summarized that the cycling regimen reduced the incidence of resistant infections or colonization from 27 to 20 isolates per 1,000 patient days (*p* = .037; Abel zur Wiesch et al., 2014). The reasons contributing to the difference might be their study included studies involving hematology patients (Chong et al., 2013) and neonatal patients (Toltzis et al., 2002). Additionally, one article that did not use a typical cycling strategy, which restricted gentamicin and tobramycin and used more amikacin at the same time, due to gram‐negative bacilli appearing highly resistant to both antibiotics, but then reintroduced gentamicin after it restored sensitivity to gram‐negative bacilli (Gerding et al., 1991). The common definition of antibiotic cycling is that several antibiotics are used in turns according to pre‐made schedule. However, the regimen of the latter study was a limitation of non‐sensitivity antibiotics; therefore, we thought it was not a typical cycling strategy.

Similarly, the meta‐analysis by Baur and colleagues has well summarized the effect of various antibiotic stewardship measures on the acquisition of antibiotic‐resistant microorganisms (Baur et al., 2017). However, the meta‐analysis included only three articles related to a cycling regimen, and one of them used limiting non‐sensitive antibiotics for controlling resistant infection that was different from the cycling strategy (Takesue et al., 2010), which might question the accuracy of conclusions.

### Antibiotic Heterogeneity

We found that the cycling strategy and mixing strategy showed more advantages than the baseline period. The favorable factors behind this might be the change of antibiotic heterogeneity (Beardmore, Peña‐Miller, Gori, & Iredell, 2017; Bonhoeffer, Wiesch, & Kouyos, 2010; Reluga, 2005). There was some difference between the baseline and mixing periods. Antibiotics were prescribed by physicians according to their own habits in a baseline period, but in a period of mixing strategy antibiotics were alternatively prescribed according to the order of admission.

The antibiotic homogeneity index (AHI), also called the Peterson index, is calculated as following formula: 1 − {*n*/[2 × (*n* − 1)]} × ∑|*A_i_* − *B_i_*|, where n is the number of considered antibiotics in this equation, *A_i_* is the proportion when all antibiotics in a given period were used in the same proportion, and Bi is the actual proportion of antimicrobials in the study (Plüss‐Suard, Pannatier, Kronenberg, Mühlemann, & Zanetti, 2013; Sandiumenge et al., 2006). The value of the AHI ranges from 0 to 1, with 0 indicating no heterogeneity and 1 indicating complete heterogeneity. The study by Sandiumenge found that the diversity of antibiotic prescriptions was higher in the mixing period than in the cycling period (Sandiumenge et al., 2006), and the cycling period was associated with a greater risk of acquisition of resistant infections (RR = 1.495, 95% CI 1.090–2.050). A large‐sample study analyzed data from 20 acute care hospitals in Switzerland and suggested a negative correlation between the drug resistance rate and diversity of using antibiotics (coefficient = −0.52; *p* < .05) (Plüss‐Suard et al., 2013).

### About Methodological Quality

The time sequence of the cycling and control periods seem to have a significant impact on the incidence of resistant microorganisms. The different antibiotic stewardship approaches, cycling or mixing, were randomly assigned to different ICUs in two prospective multicenter studies, and the results did not show any significant difference between cycling and mixing (control) periods by an analysis of the combined data (RR = 1.044, 95% CI 0.876–1.245, *p* = .632; Martínez et al., 2006; Van Duijn et al., 2018). Nine out of 12 studies with the cycling period after the control period showed that the cycling strategy substantially reduced the incidence of resistant bacteria (RR = 0.727, 95% CI 0.565–0.935, *p* = .013). Only one study (Sandiumenge et al., 2006) with cycling before the mixing period showed results in favor of the mixing strategy (RR = 1.495, 95% CI 1.090–2.050, *p* = .013). It means that the antibiotic stewardship measures conducted in the latter period presented more effective approaches than those performed in the previous period from available studies so far. Unfortunately, the measures for controlling nosocomial infections might be strengthened along with the progress of a study. For instance, an educational program that included controlling catheter‐related infections was implemented in the middle of the study (Sandiumenge et al., 2006), while measures of controlling ventilator‐associated pneumonia (VAP) were strengthened during Warren’s study (Warren et al., 2004). Non‐research arms, such as environmental cleaning, isolation, hand hygiene, chlorhexidine bathing, and preventing VAP, might become confounders in antibiotic stewardship studies. Therefore, we assume that multicenter, cluster‐randomized and crossover designs are a good way to limit these confounders.

## Conclusions

The cycling strategy and the mixing strategy seem to be effective measures to control the prevalence of antibiotic‐resistant bacteria in comparison with no specific antibiotic stewardship in the baseline period. Both of them take effect primarily through antibiotic heterogeneity and limiting antimicrobial consumption to increase the chance of natural loss of resistance genes. We cannot draw a definitive conclusion because these pooled results were influenced by other measures controlling nosocomial infections and deficiencies in methodology.

**Linking Evidence to Action**

- The cycling strategy and mixing strategy showed better than uncontrolled usage of antibiotics in the baseline period, but multiple confounders influence us to interpret this conclusion cautiously.
- The effect of cycling strategy and mixing strategy was better than that of the baseline period, mainly because of increasing antibiotic heterogeneity.
- The defined daily dose is an important indicator reflecting antibiotic selection pressure on microorganism flora. It should be considered in future antibiotic stewardship studies.
- Medical staff should be aware of the importance of avoiding low sensitivity antibiotics for empirical use.

## Supporting information


**Table S1**. The Search Strategy for Cycling Antibiotic Use
**Table S2**. The National Institutes of Health’s Quality Assessment Tool for Before‐After (Pre–Post) Studies With No Control Group
**Table S3**. The PRISMA Checklist for Antibiotic‐Cycling Strategy
**Table S4**. Characteristics of the Studies Included in the Meta‐analysis.
**Figure S1**. Forest plot evaluating the effect of antibiotic cycling on the incidence of different types of antibiotic‐resistant bacteria.
**Figure S2**. Forest plots evaluating the effect of cycling strategy on nosocomial infection and VAP.
**Figure S3**. The funnel plot for publication bias.
