# Supplementary material for: The Effect of Antibiotic‐Cycling Strategy on Antibiotic‐Resistant Bacterial Infections or Colonization in Intensive Care Units: A Systematic Review and Meta‐Analysis
Source: Worldviews Evid Based Nurs. 2020 Aug 26;17(4):319–28. doi: 10.1111/wvn.12454 (PMC7496894; doi:10.1111/wvn.12454)
Supplement: Supplementary file 1 — Table S1. The Search Strategy for Cycling Antibiotic Use Table S2. The National Institutes of Health’s Quality Assessment Tool for Before‐After (Pre–Post) Studies With No Control Group Table S3. The PRISMA Checklist for Antibiotic‐Cycling Strategy Table S4. Characteristics of the Studies Included in the Meta‐analysis. Figure S1. Forest plot evaluating the effect of antibiotic cycling on the incidence of different types of antibiotic‐resistant bacteria. Figure S2. Forest plots evaluating the effect of cycling strategy on nosocomial infection and VAP. Figure S3. The funnel plot for publication bias. [file WVN-17-319-s001.docx]

**Table S1.** The Search Strategy for Cycling Antibiotic Use

|  |
| --- |
| 1. antimicrobial*resistan*[Title/Abstract] OR antibiotic*resistan*[Title/Abstract] OR drug resistan*[MeSH Terms] OR multidrug*resistan*[Title/Abstract] OR multi*drug*resistan*[Title/Abstract] OR MDR[Title/Abstract] OR pandrug resistan*[Title/Abstract] OR pan*drug resistan*[Title/Abstract] OR carbapenem*resistan*[Title/Abstract] OR carbapenemase[Title/Abstract] OR extended-spectrum β-lactamase[Title/Abstract] OR extended-spectrum beta-lactamase[Title/Abstract] OR ESBL[Title/Abstract] OR Acinetobacter baumannii[Title/Abstract] OR A. baumannii[Title/Abstract] OR Clostridium difficile[Title/Abstract] OR C. difficile[Title/Abstract] OR methicillin-resistant staphylococcus aureus[Title/Abstract] OR MRSA[Title/Abstract] OR pseudomonas aeruginosa[Title/Abstract] OR P. aeruginosa[Title/Abstract] OR stenotrophomonas maltophilia[Title/Abstract] OR S. maltophilia[Title/Abstract] OR vancomycin resistant enterococcus[Title/Abstract] OR VRE[Title/Abstract] 2. cycling[Title/Abstract] OR rotat*[Title/Abstract] OR schedule*[Title/Abstract] 3. #1 AND #2 AND 3# |

**Table S2.** The National Institutes of Health’s Quality Assessment Tool for Before-After (Pre–Post) Studies With No Control Group

| **Criteria** | **Yes** | **No** | **Other (CD, NR, NA)*** |
| --- | --- | --- | --- |
| 1. Was the study question or objective clearly stated? |  |  |  |
| 2. Were eligibility/selection criteria for the study population pre-specified and clearly described? |  |  |  |
| 3. Were the participants in the study representative of those who would be eligible for the test/service/intervention in the general or clinical population of interest? |  |  |  |
| 4. Were all eligible participants that met the pre-specified entry criteria enrolled? |  |  |  |
| 5. Was the sample size sufficiently large to provide confidence in the findings? |  |  |  |
| 6. Was the test/service/intervention clearly described and delivered consistently across the study population? |  |  |  |
| 7. Were the outcome measures pre-specified, clearly defined, valid, reliable, and assessed consistently across all study participants? |  |  |  |
| 8. Were the people assessing the outcomes blinded to the participants' exposures/interventions? |  |  |  |
| 9. Was the loss to follow-up after baseline 20% or less? Were those lost to follow-up accounted for in the analysis? |  |  |  |
| 10. Did the statistical methods examine changes in outcome measures from before to after the intervention? Were statistical tests done that provided p values for the pre-to-post changes? |  |  |  |
| 11. Were outcome measures of interest taken multiple times before the intervention and multiple times after the intervention (i.e., did they use an interrupted time-series design)? |  |  |  |
| 12. If the intervention was conducted at a group level (e.g., a whole hospital, a community, etc.) did the statistical analysis take into account the use of individual-level data to determine effects at the group level? |  |  |  |

Note: *CD = cannot determine; NA = not applicable; NR = not reported;

**Table S3.** The PRISMA Checklist for Antibiotic-Cycling Strategy

| **Section/topic** | **#** | **Checklist item** | **Reported on page #** |
| --- | --- | --- | --- |
| **TITLE** | | |  |
| Title | 1 | Identify the report as a systematic review, meta-analysis, or both. | 1 |
| **ABSTRACT** | | |  |
| Structured summary | 2 | Provide a structured summary including, as applicable: background; objectives; data sources; study eligibility criteria, participants, and interventions; study appraisal and synthesis methods; results; limitations; conclusions and implications of key findings; systematic review registration number. | 2 |
| **INTRODUCTION** | | |  |
| Rationale | 3 | Describe the rationale for the review in the context of what is already known. | 2 |
| Objectives | 4 | Provide an explicit statement of questions being addressed with reference to participants, interventions, comparisons, outcomes, and study design (PICOS). | 3 |
| **METHODS** | | |  |
| Protocol and registration | 5 | Indicate if a review protocol exists, if and where it can be accessed (e.g., Web address), and, if available, provide registration information including registration number. | 3 |
| Eligibility criteria | 6 | Specify study characteristics (e.g., PICOS, length of follow-up) and report characteristics (e.g., years considered, language, publication status) used as criteria for eligibility, giving rationale. | 4-5 |
| Information sources | 7 | Describe all information sources (e.g., databases with dates of coverage, contact with study authors to identify additional studies) in the search and date last searched. | 4-5 |
| Search | 8 | Present full electronic search strategy for at least one database, including any limits used, such that it could be repeated. | 4 |
| Study selection | 9 | State the process for selecting studies (i.e., screening, eligibility, included in systematic review, and, if applicable, included in the meta-analysis). | 4-5 |
| Data collection process | 10 | Describe method of data extraction from reports (e.g., piloted forms, independently, in duplicate) and any processes for obtaining and confirming data from investigators. | 5 |
| Data items | 11 | List and define all variables for which data were sought (e.g., PICOS, funding sources) and any assumptions and simplifications made. | 5 |
| Risk of bias in individual studies | 12 | Describe methods used for assessing risk of bias of individual studies (including specification of whether this was done at the study or outcome level), and how this information is to be used in any data synthesis. | 6 |
| Summary measures | 13 | State the principal summary measures (e.g., risk ratio, difference in means). | 6 |
| Synthesis of results | 14 | Describe the methods of handling data and combining results of studies, if done, including measures of consistency (e.g., I^2^) for each meta-analysis. | 6 |
| Risk of bias across studies | 15 | Specify any assessment of risk of bias that may affect the cumulative evidence (e.g., publication bias, selective reporting within studies). | 6 |
| Additional analyses | 16 | Describe methods of additional analyses (e.g., sensitivity or subgroup analyses, meta-regression), if done, indicating which were pre-specified. | 6 |
| **RESULTS** |  |  |  |
| Study selection | 17 | Give numbers of studies screened, assessed for eligibility, and included in the review, with reasons for exclusions at each stage, ideally with a flow diagram. | 7 |
| Study characteristics | 18 | For each study, present characteristics for which data were extracted (e.g., study size, PICOS, follow-up period) and provide the citations. | 7 |
| Risk of bias within studies | 19 | Present data on risk of bias of each study and, if available, any outcome level assessment (see item 12). | 7 |
| Results of individual studies | 20 | For all outcomes considered (benefits or harms), present, for each study: (a) simple summary data for each intervention group (b) effect estimates and confidence intervals, ideally with a forest plot. | 7 |
| Synthesis of results | 21 | Present results of each meta-analysis done, including confidence intervals and measures of consistency. | 7-8 |
| Risk of bias across studies | 22 | Present results of any assessment of risk of bias across studies (see Item 15). | 9 |
| Additional analysis | 23 | Give results of additional analyses, if done (e.g., sensitivity or subgroup analyses, meta-regression [see Item 16]). | 9 |
| **DISCUSSION** |  |  |  |
| Summary of evidence | 24 | Summarize the main findings including the strength of evidence for each main outcome; consider their relevance to key groups (e.g., healthcare providers, users, and policy makers). | 9-12 |
| Limitations | 25 | Discuss limitations at study and outcome level (e.g., risk of bias), and at review-level (e.g., incomplete retrieval of identified research, reporting bias). | 13 |
| Conclusions | 26 | Provide a general interpretation of the results in the context of other evidence, and implications for future research. | 14 |
| **FUNDING** |  |  |  |
| Funding | 27 | Describe sources of funding for the systematic review and other support (e.g., supply of data); role of funders for the systematic review. | 15 |

**Table S4.** Characteristics of the Studies Included in the Meta-analysis.

| Author | Year | Country | Study Period | Prospec-  tive | Setting | Infection or Colonization | Control Period | Cycling Duration (month) | Cycling Length (month) | Quality (points) | Antibiotics in the Cycling Duration |
| --- | --- | --- | --- | --- | --- | --- | --- | --- | --- | --- | --- |
| Cobos-  Trigueros | 2016 | Spain | 2006-2008 | yes | medical | infection &  colonization | mixing | 4.5 | 1.5 | 9 | meropenem, ceftazidime or piperacillin- tazobactam, ciprofloxacin/levofloxacin |
| Evans | 2005 | USA | 1999-2001 | yes | surgical | infection | baseline | 12 | 3 | 7 | carbapenem, cefepime, ciprofloxacin, piperacillin-tazobactam |
| Gruson | 2000 | France | 1995-1998 | yes | medical | infection | baseline | 24 | 1 | 6 | cefepime, piperacillin-tazobactam, imipenem, ticarcillin-clavulanic |
| Hedrick | 2008 | USA | 2000-2002 | yes | medical | infection &  colonization | baseline | 18 | 3 | 6 | cefepime, ciprofloxacin, piperacillin-tazobactam, carbapenem |
| Martínez | 2006 | Spain | 2001-2002 | yes | medical | infection &  colonization | mixing | 8 | 1 | 7 | cephalosporin, ﬂuoroquinolone, carbapenem, piperacillin-tazobactam |
| Nijssen | 2010 | Nether-  lands | 2001-2002 | no | mixed | colonization | baseline | 3 | 0.25 | 6 | ceftriaxone, amoxicillin-clavulanic, fluoroquinolone |
| Raineri | 2010 | Italy | 2006-2007 | no | mixed | infection | baseline | 12 | 3 | 8 | piperacillin-tazobactam, quinolone, carbapenem, cefepime/ceftazidime |
| Raymond | 2001 | USA | 1997-1999 | yes | mixed | infection | baseline | 12 | 3 | 9 | ciproﬂoxacin+-clindamycin, piperacillin-tazobactam, carbapenem, cefepime+-clindamycin |
| Sandiu-  menge | 2006 | Spain | 2000-2003 | yes | mixed | infection &  colonization | mixing | 12 | 4 | 9 | carbapenem, cephalosporin, piperacillin-tazobactam |
| Smith | 2008 | USA | 1997-2003 | no | mixed | infection | baseline | 24 | 3 | 7 | linezolid, vancomycin |
| van Duijn | 2018 | Nether-  lands | 2011-2014 | yes | mixed | colonization | mixing | 18 | 1.5 | 10 | cephalosporin, piperacillin-tazobactam, carbapenem |
| Warren | 2004 | USA | 2000-2002 | no | medical | infection &  colonization | baseline | 24 | 3.4 | 5 | cephalosporin, ﬂuoroquinolone, carbapenem, piperacillin-tazobactam |

Note: mixed: including medical and surgical patients; mixing: the first-line antibiotic will alternate in consecutive patients according to a pre-established protocol; “+-”: with or without an antibiotic.

**Figure S1.** Forest plot evaluating the effect of antibiotic cycling on the incidence of different types of antibiotic-resistant bacteria.


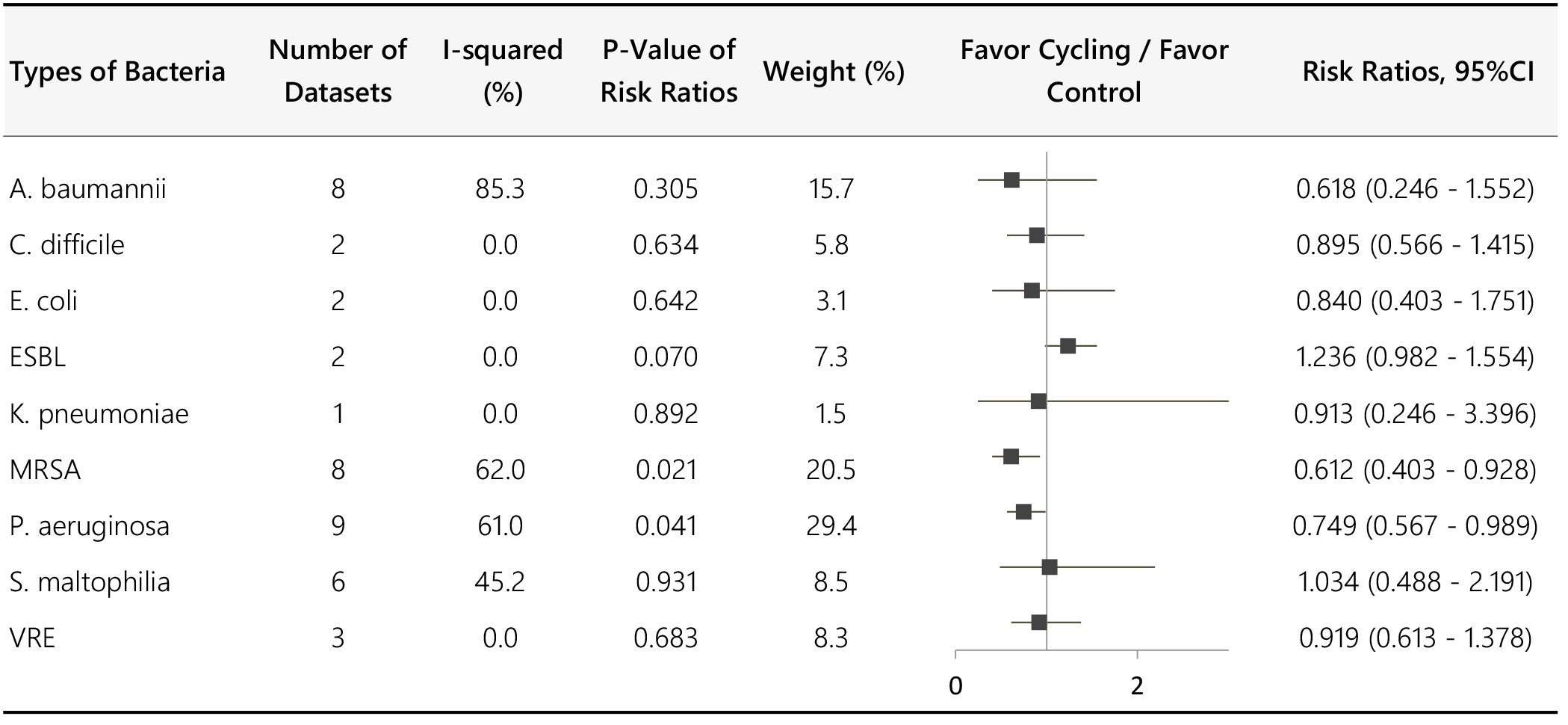


Note: *A. baumannii* = *Acinetobacter baumannii*; *C. difficile* = *Clostridium difficile*; *E. coli* = *Escherichia coli*; ESBL = extended-spectrum beta-lactamase; *K. pneumoniae* = *Klebsiella pneumoniae*; MRSA = methicillin-resistant *Staphylococcus aureus*; *P. aeruginosa* = *Pseudomonas aeruginosa*; *S. maltophilia* = *Stenotrophomonas maltophilia*; VRE = vancomycin-resistant *Enterococcus*; RR = risk ratio.

**Figure S2.** Forest plots evaluating the effect of cycling strategy on nosocomial infection and VAP.


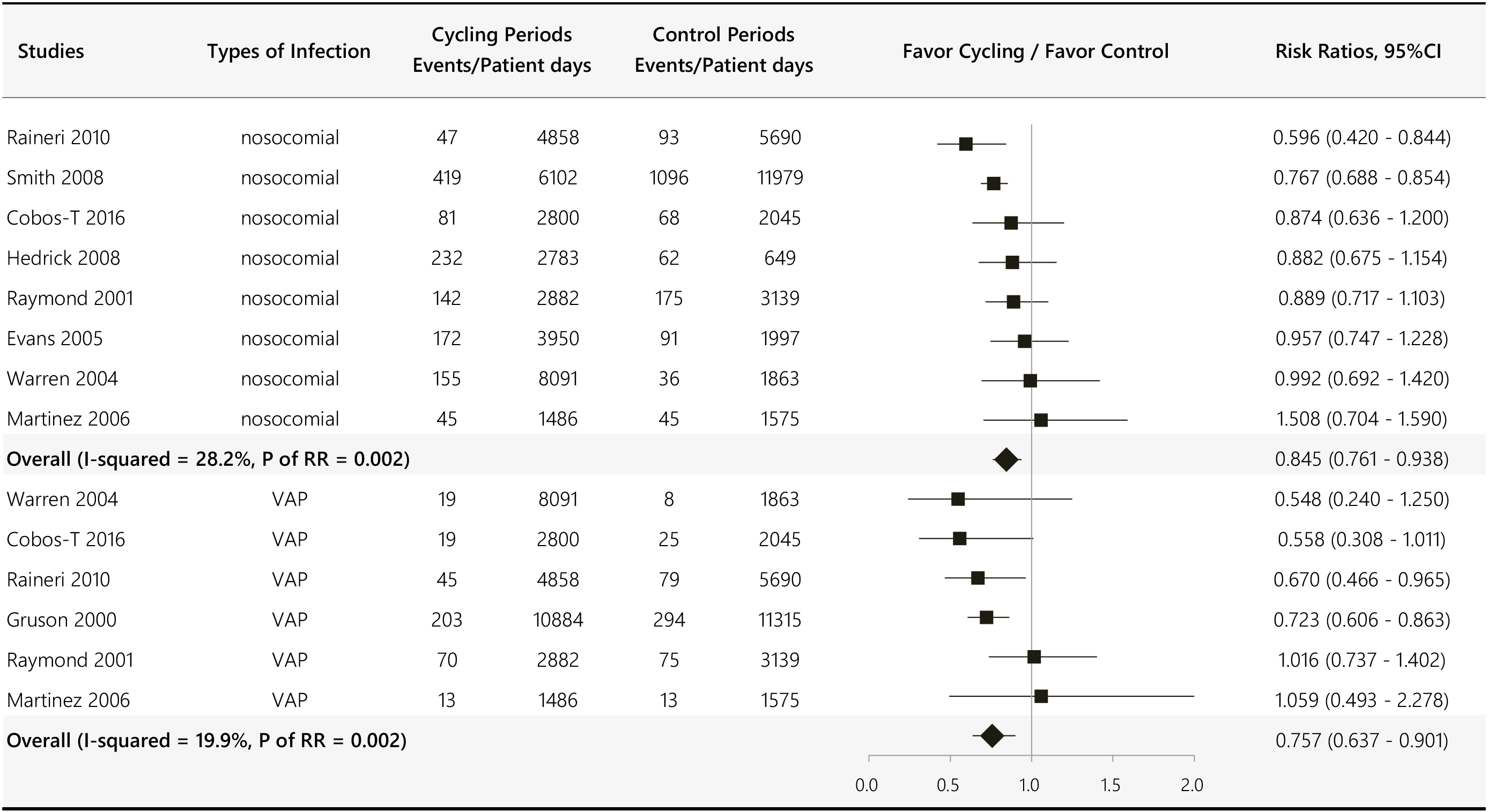


**Figure S3.** The funnel plot for publication bias.
